# A haplotype of polymorphisms in *ASE-1*, *RAI *and *ERCC1 *and the effects of tobacco smoking and alcohol consumption on risk of colorectal cancer: a danish prospective case-cohort study

**DOI:** 10.1186/1471-2407-8-54

**Published:** 2008-02-20

**Authors:** Rikke D Hansen, Mette Sørensen, Anne Tjønneland, Kim Overvad, Håkan Wallin, Ole Raaschou-Nielsen, Ulla Vogel

**Affiliations:** 1 National Research Centre for the Working Environment, Lersø Parkalle 105, 2100 Copenhagen, Denmark; 2 Institute of Cancer Epidemiology, Danish Cancer Society, Strandboulevarden 49, 2100 Copenhagen, Denmark; 3 Department of Clinical Epidemiology, Aalborg Hospital, Stengade 10, Postbox 561 Aarhus University Hospital, 9000 Aalborg, Denmark; 4 Department of Science, Systems and Models, University of Roskilde, 4000 Roskilde, Denmark

## Abstract

**Background:**

Single nucleotide polymorphisms (SNPs) are the most frequent type of genetic variation in the human genome, and are of interest for the study of susceptibility to and protection from diseases. The haplotype at chromosome 19q13.2-3 encompassing the three SNPs *ASE-1 *G-21A, *RAI *IVS1 A4364G and *ERCC1 *Asn118Asn have been associated with risk of breast cancer and lung cancer. Haplotype carriers are defined as the homozygous carriers of *RAI *IVS1 A4364G^A^, *ERCC1 *Asn118Asn^T ^and *ASE-1 *G-21A^G^. We aimed to evaluate whether the three polymorphisms and the haplotype are associated to risk of colorectal cancer, and investigated gene-environment associations between the polymorphisms and the haplotype and smoking status at enrolment, smoking duration, average smoking intensity and alcohol consumption, respectively, in relation to risk of colorectal cancer.

**Methods:**

Associations between the three individual polymorphisms, the haplotype and risk of colorectal cancer were examined, as well as gene-environment interaction, in a Danish case-cohort study including 405 cases and a comparison group of 810 persons. Incidence rate ratio (IRR) were estimated by the Cox proportional hazards model stratified according to gender, and two-sided 95% confidence intervals (CI) and p-values were calculated based on robust estimates of the variance-covariance matrix and Wald's test of the Cox regression parameter.

**Results:**

No consistent associations between the three individual polymorphisms, the haplotype and risk of colorectal cancer were found. No statistically significant interactions between the genotypes and the lifestyle exposures smoking or alcohol consumption were observed.

**Conclusion:**

Our results suggest that the *ASE-1 *G-21A, *RAI *IVS1 A4364G and *ERCC1 *Asn118Asn polymorphisms and the previously identified haplotype are not associated with risk of colorectal cancer. We found no evidence of gene-environment interaction between the three polymorphisms and the haplotype and smoking intensity and alcohol consumption, respectively, in relation to the risk of colorectal cancer.

## Background

SNPs in DNA repair genes have been associated to risk of several types of cancer, reviewed in [1]. Combining of neighbouring SNPs into haplotypes may increase the association with disease. We have previously identified a haplotype at chromosome 19q13.2-3 encompassing three SNPs in the genes *ERCC1 *(excision repair cross complementary group1)*, ASE-1 *(antisense *ERCC1*, alias *CD3EAP*) and *RAI *(RelA-associated inhibitor, alias *iASPP *or *PPP1R13L*), strongly associated with risk of post-menopausal breast cancer [2] and lung cancer [3,4], and a recent Norwegian study has reported a possible association with risk of colorectal cancer among women [5]. Thus, the haplotype seems to be involved in a mechanism affecting various cancer forms. The previously identified haplotype consists of three marker SNPs: *RAI *IVS1 A4364G, *ERCC1 *Asn118Asn and *ASE-1 *G-21A, of which haplotype carriers are defined as the homozygous carriers of *RAI *IVS1 A4364G^A^, *ERCC1 *Asn118Asn^T ^and *ASE-1 *G-21A^G^.

*RAI *encodes a specific inhibitor of the RelA subunit (p65) in the NF-κB transcription factor [6], which participates in inflammatory responses [7] and as an regulator of apoptosis [8]. The regulator function of NF-κB, as an inhibitor or an activator of apoptosis, may depend on the ratio between the two NF-κB subunits RelA and c-Rel: An over-expression of RelA inhibits apoptosis, while an over-expression of c-Rel enhances apoptosis [8]. Inhibition of RAI synthesis has been shown to inhibit apoptosis [9]. In human colorectal adenomas and adenocarcinomas a constitutively increased expression of RelA/NF-κB protein have been observed [10,11]. The increase was not detected in healthy colorectal mucosa [10]. It was recently shown that *RAI *expression is increased in both adenoma and adenocarcinoma tissue compared to tissue from normal colonic mucosa in the same person [12] indicating that high *RAI *expression occurs very early in the malignant transformation. However, contradictory results have been published as to whether the increased expression levels inhibit or promote apoptosis [9,13]. The polymorphisms in *ERCC1 *and in particular *RAI *are strongly associated with risk of basal cell carcinoma [14,15].

*ERCC1 *encodes a subunit of the nucleotide excision repair (NER) complex, which is required for the incision step of NER [16]. For colorectal and lung cancer patients the mRNA levels and the expression of the ERCC1 protein are associated with the response to platinum-based chemotherapeutic drugs with direct impact on cancer patient survival. The level of response may be related to the *ERCC1 *Asn118Asn polymorphism [17-19], and the *ERCC1 *C8092A polymorphism as well, for lung cancer patients [20]. These epidemiological results are not entirely consistent, but they may indicate that the polymorphism predicts DNA repair capacity.

*ASE-1 *encodes a nucleolar protein, and is positioned in an anti-sense orientation to and overlaps with the gene for *ERCC1*. It is possibly involved with the RNA polymerase I transcription complex [21]. The exact role of *ASE-1 *and whether the overlapping between *ERCC1 *and *ASE-1 *gene has biological relevance are unknown.

In studies of Caucasians, the haplotype encompassing the three SNPs: *ERCC1 *Asn118Asn, *ASE-1 *G-21A and *RAI *IVS1 A4364G was strongly associated to risk of post-menopausal breast cancer [2] and lung cancer [3] among women less than 55 years of age when diagnosed with their first cancer. Female homozygous carriers of the haplotype were at a 7.02-fold (CI: 1.88–26.18) higher risk of lung cancer compared to female non-carriers of the homozygous haplotype, while the haplotype was associated to risk of breast cancer with an RR of 9.50 (CI: 2.21–40.79) among female homozygous carriers of the haplotype, compared to women not carrying the homozygous haplotype. An association was not detected in the older age groups (55–60 years and > 60 years when diagnosed with breast cancer) [2].

The mechanisms underlying the possible association between alcohol consumption and tobacco smoking and risk of colorectal cancer are unknown. Smoking has consistently been associated with increased risk for adenomas/polyps in colon and rectum [22]. Following tobacco smoking and intake of alcohol, DNA adducts are formed by metabolites of the carcinogens polycyclic aromatic hydrocarbons, aromatic amines, nitrosamines, and ethanol [23,24]. The adducts are not only located in airway tissue or the upper gastrointestinal tract, but are also found in e.g. white blood cells, bladder and cervical tissue [25-27], indicating that systemic circulation of the carcinogens takes place. Thus, DNA lesions following tobacco smoking and alcohol consumption combined with a low DNA repair capacity or deficiency of triggering apoptosis in colonocytes may possibly lead to malignant transformation of the cells.

To our knowledge only two studies have been reported on gene-environment interactions with the predefined haplotype: A Norwegian case-control study on colorectal adenomas and adenocarcinomas [5] and our prospective case-cohort study on lung cancer [4]. No interaction was observed between the haplotype and cigarette smoking and intake of alcohol, respectively, in relation to risk of colorectal adenomas or adenocarcinomas [5]. In the lung cancer study, a stronger adverse association between smoking and risk of lung cancer were observed among women with a high smoking intensity (> 20 g tobacco/day) carrying the homozygous high risk haplotype [4], than among female non-carriers of the homozygous haplotype with the same smoking intensity. An interaction between alcohol consumption and the haplotype was also found: Male carriers of the haplotype had a higher risk of lung cancer, whereas no association was found among men who were not carrying the homozygous haplotype.

In the present study, we hypothesized that female homozygous carriers of the previously identified haplotype have higher risk of colorectal cancer compared to individuals not carrying the homozygous haplotype, [5]. We expect that the haplotype or individual SNPs are linked to a biologically effective polymorphism. *RAI *is an obvious candidate gene, since it has been shown to be required for induction of apoptosis [28], possibly through interaction with the *p53 *gene [13]. Furthermore, we hypothesized the haplotype to interact with environmental factors such as smoking and alcohol consumption, in relation to risk of colorectal cancer as previously observed for lung cancer.

## Methods

In 1993–1997, a total of 160,725 individuals were invited to participate in the Danish prospective "Diet, Cancer and Health" (DCH) study, the inclusion criteria being age 50–64 years, born in Denmark and with no prior cancer diagnosis at the time of inclusion. In total, 57,053 participants accepted the invitation and were enrolled in the cohort [28]. At enrolment detailed information on diet, smoking habits, lifestyle, weight, height, reproduction, medical treatment, and other socio-economic characteristics and environmental exposures were collected. Moreover, blood, urine, fat tissue and other biological material was sampled and stored at -150°C. All participants gave informed consent. DCH and the present sub-study were approved by the Regional Ethical Committees on Human Studies in Copenhagen and Aarhus and by the Danish Data Protection Agency. Among the cohort members included in this study, 405 incident cases of colorectal cancer were identified, 184 women and 221 men, in the files of the nationwide Danish Cancer Registry [29], diagnosed between 1994 and 2003. Within the cohort we defined a sub-cohort consisting of 368 women and 442 men who were randomly selected. Cases and sub-cohort were matched on gender. Blood samples were available for 397 cases and 800 members of the sub-cohort. Information on smoking habits and alcohol consumption were available for all participants in the present study. Characteristics of the cases and the sub-cohort are shown in Table 1.

**Table 1:** Characteristics of the study population nested within the Danish cohort Diet, Cancer and Health.

	Cases	Sub-cohort
*Total*^a^	405 (100)	810 (100)
*Gender*^a^		
Men	221 (55)	442 (54)
Women	184 (45)	368 (46)
*Age at inclusion*^b^	58 (51–64)	56 (50–63)
*Age at diagnosis*^b^	62 (54–70)	-
*Alcohol intake*, g/day^b^		
Total	13 (1–69)	12 (1–63)
Men	23 (2–88)	19 (1–77)
Women	8 (0–41)	8 (1–39)
*Smoking status at inclusion*^a^		
Never	123 (30)	272 (33)
Former	122 (30)	252 (31)
Present	160 (40)	286 (36)
*Smoking intensity*, g tobacco/day^b^		
Total	15 (4–37)	15 (2–35)
Men	19 (4–41)	18 (2–40)
Women	12 (3–22)	12 (2–23)
*Smoking duration*, years^b^		
Total	34 (8–47)	32 (6–46)
Men	34 (8–48)	32 (8–48)
Women	34 (7–45)	32 (4–44)

a) Number (%)

b) Median (5–95% percentiles)

DNA was isolated from frozen blood samples as described [30]. All genotypes were determined by endpoint reading on a Sequence Detection System ABI 7500 (Applied Biosystems, Nærum, Denmark). The case-control status of the samples was blinded when analysing the DNA in the laboratory. Controls with known genotypes were included in each run and repeated genotyping of a random 10% subset yielded 100% identical genotypes.

Primers and probes for *ERCC1 *Asn118Asn (C/T) (rs #3177700), *ASE-1 *G-21A (rs #967591) and *RAI *A4364G (rs #1970764) genotyping were as previously described [4,31].

Five μL reactions contained 100 nM of each probe, 900 nM primers, 20–50 ng DNA and 1x Mastermix (Applied Biosystems, Nærum, Denmark). The polymorphisms were analysed for 40 cycles at 15s at 94°C, and an elongation step performed at 62°C (*ASE-1*), 62°C (*RAI*) and 63°C (*ERCC1*) for 60s.

The *ERCC1 *Asn118Asn genotyping was unsuccessful for 7 samples, whereas 2 were unsuccessful for *ASE-1 *G-21A genotyping, and 8 for *RAI *A4364G.

The data were sampled according to the case-cohort design and the unweighted case-cohort approach was used for analysis [32]. Incidence rate ratios (IRR) for colorectal cancer were estimated by the Cox proportional hazards model. All the analyses were stratified according to gender. Age was the underlying time axis. The analyses were corrected for delayed entry, thereby considering risk only from the person's age at enrolment in the cohort. We calculated two-sided 95% confidence intervals (CI) and p-values based on robust estimates of the variance-covariance matrix [33] and Wald's test of the Cox regression parameter, that is, on the log rate ratio scale.

The genotype distributions of the polymorphisms were checked among the sub-cohort to verify Hardy-Weinberg equilibrium. We estimated IRR for the heterozygous and the homozygous variant, respectively, compared with the homozygous wild type. These estimates were supplemented with a trend test where the IRR is assumed to increase/decrease log-linearly with the number of variant type alleles. We analyzed data separately for each gender and each of two age groups (≤ 60 years and > 60 years) and separately according to topology of the cancer (colon or rectum, right- or left-sided segment of colon).

Smoking intensity was calculated as the average of the self-reported smoking intensity for the age intervals 20–29, 30–39, 40–49 and 50–59 years and reported smoking status, smoking duration and alcohol consumption refer to the time of enrolment into the cohort study. Former smokers were defined as the participants who have been smoking regularly, at least one cigarette, pipe, cheroot or cigar daily, for at least one year, but who were not smoking daily at the time of enrolment. Smoking intensity for former smokers were calculated as the smoking intensity for each age intervals subtracted the periods of not smoking.

For each of the continuous variables, the hypothesis of a linear association was evaluated using a linear spline with three boundaries, placed at the quartiles among cases, as covariates in the Cox model [34]. The linearity was evaluated graphically and by a numerical test using the likelihood ratio test statistic to compare the model assuming linearity with the linear spline model. All variables were found linearly associated with colorectal cancer.

Interactions between the genotypes and each of the environmental factors, smoking status, smoking duration, average smoking intensity and alcohol consumption, were analysed by estimating separate associations for each genotype (homozygous wild type, heterozygous type and homozygous variant type). We tested the null hypothesis that the effect of the environmental factor was the same for the three genotypes. All models on gene-environment interactions were adjusted for smoking status (ever/never) or alcohol intake (yes/no). Thus, the effect of smoking intensity and duration were estimated among smokers only, and the effect of alcohol intake was estimated among alcohol consumers only. In the Cox regression model, we stratified according to gender allowing separate underlying risk for men and women, respectively. Hence, adjustment for HRT use in the analyses affects the risk estimates for women only. We used SAS PROC PHREG (version 8.2) for the estimations.

An 80% power required IRRs between 1.3 and 1.4 per variant allele for the three polymorphisms in the analyses of a linear trend at a significance level of 5%. A similar power required an IRR of 1.5 for the homozygous haplotype versus non-carriers of the homozygous haplotype. The power calculations were made on the basis of the allele frequencies and number of participants in the present study.

## Results

Fifty-two percent of the cases were diagnosed with colon cancer, 45% with rectal cancer, and in 3% of the cases the topography was not specified. Among cases, 96% had a diagnosis of adenocarcinomas, 2% of carcinoid tumor and 2% of various other histological subtypes. The distribution of cancer location was comparable for men and women, data not shown. The median consumption of alcohol was comparable between cases and sub-cohort members (Table 1). The proportion of present smokers was higher among cases than among members of the sub-cohort. Smoking intensity was comparable between cases and members of the sub-cohort, while smoking duration among male and female cases, respectively, were longer than among members of the sub-cohort. Smoking intensity was substantially higher among men than among women. In addition, the alcohol consumption was higher among men than women (Table 1).

The genotype distributions were in Hardy-Weinberg equilibrium among the sub-cohort members for all three polymorphisms both before and after gender separation, results not shown. The allelic frequencies of the variant allele for *ASE-1 *G-21A (0.17), *RAI *IVS1 A4364G (0.19), and *ERCC1 *Asn118Asn (0.36) were similar to the frequencies of alleles found in previous studies of Caucasians [3,5,31]. The genotype distributions were comparable between cases and members of the sub-cohort, results not shown.

Overall, there were no statistically significant associations between the three polymorphisms and risk of colorectal cancer, either among men or women (Table 2). The association between the haplotype and risk of colorectal cancer did not differ statistically significant between men and women (P = 0.16). However, women carrying the high risk haplotype seemed to be at higher risk of colorectal cancer with an IRR of 1.35 (0.84–2.16), compared with female non-carriers of the homozygous haplotype. The association between the three polymorphisms, *ASE-1 *G-21A, *RAI *IVS1 A4364G and *ERCC1 *Asn118Asn and colorectal cancer was not statistically significant different (all P > 0.25) between genders.

**Table 2:** Association between *ERCC1 *Asn118Asn, *ASE-1*G-21A, *RAI *IVS1 A4364G polymorphism and haplotype and risk of colorectal cancer

		Men				Women			
	SNP	N _cases/sub-cohort_	IRR^ab^	CI (95%)^c^	P^d^	N _cases/sub-cohort_	IRR^ab^	CI (95%)^c^	P^d^
*ERCC1 *Asn118Asn	TT	84/177	1^e^	*		84/156	1^e^	*	
	CT	100/193	1.04	0.72–1.50	0.66	66/156	0.78	0.51–1.19	0.48
	CC	34/61	1.13	0.66–1.94		27/52	0.90	0.50–1.63	
	missing	3/11				7/4			
*ASE-1 *G-21A	GG	152/299	1^e^	*		124/248	1^e^	*	
	AG	60/124	0.94	0.64–1.38	0.73	45/106	0.85	0.55–1.32	0.96
	AA	7/10	2.11	0.71–6.21		9/11	1.58	0.60–4.18	
	missing	2/9				6/3			
*RAI *IVS1 A4364G	AA	156/280	1^e^	*		107/230	1^e^	*	
	AG	56/138	0.69	0.47–1.03	0.13	61/124	1.17	0.77–1.80	0.20
	GG	6/13	0.89	0.31–2.57		8/10	1.95	0.71–5.33	
	missing	3/11							
High risk haplotype	No	165/319	1^e^	*		125/280	1^e^	*	
	Yes^f^	53/110	1.01	0.68–1.51		51/82	1.35	0.84–2.16	
	missing	3/13				8/6			

a) IRR: incidence rate ratio

b) Adjusted for average smoking intensity, intake of alcohol, fruits/vegetables, fish/poultry, red and processed meat, dietary fibres, BMI and hormone replacement therapy.

c) CI: 95% confidence interval

d) P: trend test

e) The genotype serves as reference category

f) Homozygous carriers of the haplotype compared to the rest. Homozygous carrier of the previously identified haplotype consisting of *ERCC1 *Asn118Asn^TT^, *ASE-1 *G-21A^GG ^and *RAI *IVS1 A4364G^AA^

There were no significant effects of the three polymorphisms and the haplotype in relation to risk of colorectal cancer in the topological sub-groups (left-or right sided segment of colon or rectal, results not shown), and no statistically significant effects were observed of the three polymorphisms and the high risk haplotype in relation to risk of colorectal cancer in either of the two age groups (≤ 60 years and > 60 years), results not shown. Thus, further statistical analysis includes all cases regardless of location of the cancer and age when diagnosed with colorectal cancer. The effect of smoking status at enrolment, smoking duration, average smoking intensity and intake of alcohol on colorectal cancer risk did not differ between genders, results not shown. However, since we previously have found gender-specific gene-environment interactions in the recent lung cancer study, the interaction studies on the polymorphisms and the haplotype were stratified by gender.

There were no statistically significant interactions between the haplotype and smoking intensity or alcohol consumption (Table 3), or between the *ASE-1 *G-21A, *RAI *IVS1 A4364G and *ERCC1 *Asn118Asn polymorphisms, respectively, and smoking intensity or alcohol consumption (see Additional file 1), in relation to risk of colorectal cancer. Yet, there was a tendency that smoking intensity was a risk factor for colorectal cancer among homozygous carriers of the variant *ASE-1 *G-21A allele especially among men. No effect was observed among carriers of the wild type. We observed no statistically significant interactions between the genotypes and smoking status and smoking duration at enrolment associated to risk of colorectal cancer, results not shown.

**Table 3:** Gene-environment interaction stratified by gender. Haplotype-specific effects of average smoking intensity and consumption of alcohol on the risk of colorectal cancer

Haplotype		Men				Women			
	Carrier^a^	N _cases/sub-cohort_	IRR^b^	CI (95%)^c^	P^d^	N _cases/sub-cohort_	IRR^b^	CI (95%)^c^	P^d^
Smoking intensity^e ^(per 10 g tobacco/day)	No	130/232	0.93	0.76–1.13	0.26	62/149	1.58	0.99–2.50	0.42
	Yes	45/74	1.14	0.79–1.63		31/52	1.23	0.64–2.38	
	missing	3/8				4/4			
Alcohol intake^f ^(per 10 g/day)	No	164/313	1.04	0.97–1.12	0.15	121/276	1.15	0.98–1.36	0.17
	Yes	51/104	1.20	1.00–1.43		47/80	0.87	0.61–1.25	
	missing	3/13				7/6			

a) Homozygous carriers of the haplotype compared to the rest. Homozygous carrier of the previously identified haplotype consisting of *ERCC1 *Asn118Asn^TT^, *ASE-1 *G-21A^GG ^and *RAI *IVS1 A4364G^AA^

b) IRR: incidence rate ratio

c) CI: 95% confidence interval

d) P: test for interaction

e) Risk estimates for ever smokers only. Adjusted for smoking status (present/former/never), intake of alcohol, fruits/vegetables, fish/poultry, red and processed meat, dietary fibres, BMI and hormone replacement therapy.

f) Risk estimates for alcohol consumers only. Adjusted for average smoking intensity, fruits/vegetables, fish/poultry, red and processed meat, dietary fibres, BMI and hormone replacement therapy

## Discussion

Overall, we observed no association between the previously identified haplotype and the *RAI *IVS1 A4364G, *ASE-1 *G-21A and *ERCC1 *Asn118Asn polymorphisms and risk of colorectal cancer. We did not find any interactions between the genotypes or the haplotype and the lifestyle exposures smoking intensity or alcohol consumption in relation to risk of colorectal cancer.

The present study is population based. Selection bias is unlikely because the sub-cohort used was selected as a stratified random sample from the same cohort that gave rise to the included colorectal cancer cases. The inclusion of 405 cases and a comparison group of 810 individuals provided a relatively high statistical power. For all participants, information on smoking habits and alcohol consumption was obtained at enrolment before a cancer diagnosis, which minimizes the risk for differential misclassification of these exposures between the cases and the comparison group.

In previous studies the *RAI *IVS1 A4364G polymorphism has been associated with a lower risk of basal cell carcinoma, lung cancer and post-menopausal breast cancer [2,3,15] among homozygous carriers of the variant allele. The strongest association with risk of breast cancer or basal cell carcinoma was observed among the youngest individuals [2,14,15]. The *RAI *IVS1 A4364G polymorphism was not associated with risk of testicular cancer [31]. Our results on the *ASE-1 *G-21A and *ERCC1 *Asn118Asn polymorphisms and risk of colorectal cancer are comparable to other cancer studies, where no associations have been observed between the two polymorphisms and risk of basal cell carcinoma, post-menopausal breast cancer and testicular cancer [2,31,35].

A tendency of higher risk for colorectal carcinoma, with an OR of 2.19 (CI: 0.95–5.04), was observed among Norwegian women carrying the homozygous haplotype compared to women not carrying the homozygous haplotype [5]. The results of the present study are comparable, with an IRR of 1.35 (CI: 0.84–2.16). However, in neither of the studies statistically significant gender-specific gene effects of the haplotype were observed. Previous studies on breast and lung cancer found a strong age dependent and gender-specific effect of the haplotype on risk of cancer [2,3]. A possible reason for not detecting any association between the haplotype and risk of colorectal cancer in the present study, may thus be due to the relatively high age among cases (median 62 years) when diagnosed with colorectal cancer. We were not able to estimate a risk association for the age-group younger than 55 years due to low number of cases (8 cases carrying the homozygous haplotype). When data was stratified by age with members of the youngest age group being less or equal to 60 years when diagnosed with colorectal cancer, still no statistically significant associations between the haplotype and risk of colorectal cancer were detected, results not shown.

Smoking intensity increased the risk of colorectal cancer among homozygous carriers of the variant *ASE-1 *G-21A polymorphism. The effect may very possibly be a chance finding due to low numbers of cases and members of the sub-cohort. Thus, no convincing statistical significant interactions were observed between the three genotypes and alcohol consumption or the smoking variables related with risk of colorectal cancer.

We observed no interaction between the haplotype and smoking intensity on risk of colorectal cancer. Our previous study on risk of lung cancer found a borderline significant interaction (P = 0.06) between high smoking intensity (>20 g tobacco/day) and the haplotype related with risk of lung cancer, with a 2.03-fold increased risk of lung cancer per additional 5 g tobacco/day among women carrying the haplotype, while no association were observed among female non-carriers of the haplotype with a similar smoking intensity [4]. Among women with a lower smoking intensity (≤ 20 g tobacco/day) no effect was observed of smoking intensity on risk of lung cancer among carriers or non-carriers of the homozygous haplotype. In the present study it was not possible to access the effect of a high smoking intensity among women due to a lower smoking intensity among female cases. The findings in the present study is thus similar to the above mentioned results in the lung cancer study [4] for women with a comparable low smoking intensity.

Alcohol consumption did not have a significantly different effect on carriers and non-carriers of the homozygous haplotype (P > 0.13) among men and women, respectively, in relation to risk of colorectal cancer. However, we detected an adverse effect of alcohol among male carriers of the homozygous haplotype, with an IRR of 1.20 (CI: 1.00–1.43), per 10 g alcohol intake per day. In the Norwegian study of colorectal cancer, the alcohol consumption was lower among cases than in the present study, and approximately 40% of the cases were abstainers [36]. No interaction between the high risk haplotype and alcohol intake on risk of colorectal adenomas and carcinomas was observed [5]. In our recent lung cancer study, alcohol consumption was associated with a 1.40-fold (CI: 1.13–1.74) increased risk of lung cancer per 10 g alcohol intake per day among male carriers of the homozygous haplotype [4]. The effect of alcohol was significantly different between carriers and non-carriers of the homozygous haplotype (P = 0.003). Although a similar gene-environment interaction seems not to exist for colorectal cancer, we cannot exclude a weak tendency of interaction between alcohol consumption and the homozygous high risk haplotype among men.

## Conclusion

Overall, the *ASE-1 *G-21A, *RAI *IVS1 A4364G and *ERCC1 *Asn118Asn polymorphisms and the previously identified haplotype were not associated with risk of colorectal cancer. No statistically significant interactions were observed between the high risk haplotype, the three polymorphisms and smoking intensity and alcohol consumption, respectively, in relation to the risk of colorectal cancer.

## Competing interests

The author(s) declare that they have no competing interests.

## Authors' contributions

RDH isolated the DNA for analysis, genotyped the DNA samples, made the data analysis and drafted the manuscript

MS and ORN contributed with strategies for data analysis

AT and KO brought the idea of the "Diet, Cancer and Health" study and organized it

HW and UV contributed with idea and design of the present study

All authors contributed to interpretation of the results and to the final manuscript.

## Pre-publication history

The pre-publication history for this paper can be accessed here:



## Supplementary Material

Additional File 1 Table 4 Gene-environment interaction stratified by gender. *ERCC1 *Asn118Asn, *ASE-1 *G-21A and *RAI *IVS1 A4364G genotype-specific effects of average smoking intensity and consumption of alcohol on the risk of colorectal cancer. The table presents the genotypic distribution of the three polymorphisms in the *ERCC1*, *ASE-1 *and *RAI *genes for both cases and controls, and the incidence rate ratios of colorectal cancer risk associated with the three polymorphisms. Click here for file

## References

[B1] Goode EL, Ulrich CM, Potter JD (2002). Polymorphisms in DNA repair genes and associations with cancer risk. Cancer Epidemiol Biomarkers Prev.

[B2] Nexo BA, Vogel U, Olsen A, Ketelsen T, Bukowy Z, Thomsen BL, Wallin H, Overvad K, Tjonneland A (2003). A specific haplotype of single nucleotide polymorphisms on chromosome 19q13.2-3 encompassing the gene RAI is indicative of post-menopausal breast cancer before age 55. Carcinogenesis.

[B3] Vogel U, Laros I, Jacobsen NR, Thomsen BL, Bak H, Olsen A, Bukowy Z, Wallin H, Overvad K, Tjonneland A, Nexo BA, Raaschou-Nielsen O (2004). Two regions in chromosome 19q13.2-3 are associated with risk of lung cancer. Mutat Res.

[B4] Vogel U, Sorensen M, Hansen RD, Tjonneland A, Overvad K, Wallin H, Nexo BA, Raaschou-Nielsen O (2007). Gene-environment interactions between smoking and a haplotype of RAI, ASE-1 and ERCC1 polymorphisms among women in relation to risk of lung cancer in a population-based study. Cancer Lett.

[B5] Skjelbred CF, Sabo M, Nexo BA, Wallin H, Hansteen IL, Vogel U, Kure EH (2006). Effects of polymorphisms in ERCC1, ASE-1 and RAI on the risk of colorectal carcinomas and adenomas: A case control study. BMC Cancer.

[B6] Yang JP, Hori M, Sanda T, Okamoto T (1999). Identification of a novel inhibitor of nuclear factor-kappaB, RelA-associated inhibitor. J Biol Chem.

[B7] Greten FR, Eckmann L, Greten TF, Park JM, Li ZW, Egan LJ, Kagnoff MF, Karin M (2004). IKKbeta links inflammation and tumorigenesis in a mouse model of colitis-associated cancer. Cell.

[B8] Chen X, Kandasamy K, Srivastava RK (2003). Differential roles of RelA (p65) and c-Rel subunits of nuclear factor kappa B in tumor necrosis factor-related apoptosis-inducing ligand signaling. Cancer Res.

[B9] Laska MJ, Strandbygard D, Kjeldgaard A, Mains M, Corydon TJ, Memon AA, Sorensen BS, Vogel U, Jensen UB, Nexo BA (2007). Expression of the RAI gene is conducive to apoptosis: studies of induction and interference. Exp Cell Res.

[B10] Yu HG, Yu LL, Yang Y, Luo HS, Yu JP, Meier JJ, Schrader H, Bastian A, Schmidt WE, Schmitz F (2003). Increased expression of RelA/nuclear factor-kappa B protein correlates with colorectal tumorigenesis. Oncology.

[B11] Lind DS, Hochwald SN, Malaty J, Rekkas S, Hebig P, Mishra G, Moldawer LL, Copeland EM, Mackay S (2001). Nuclear factor-kappa B is upregulated in colorectal cancer. Surgery.

[B12] Saebo M, Skjelbred CF, Nexo BA, Wallin H, Hansteen IL, Vogel U, Kure EH (2006). Increased mRNA expression levels of ERCC1, OGG1 and RAI in colorectal adenomas and carcinomas. BMC Cancer.

[B13] Bergamaschi D, Samuels Y, O'Neil NJ, Trigiante G, Crook T, Hsieh JK, O'Connor DJ, Zhong S, Campargue I, Tomlinson ML, Kuwabara PE, Lu X (2003). iASPP oncoprotein is a key inhibitor of p53 conserved from worm to human. Nat Genet.

[B14] Rockenbauer E, Bendixen MH, Bukowy Z, Yin J, Jacobsen NR, Hedayati M, Vogel U, Grossman L, Bolund L, Nexo BA (2002). Association of chromosome 19q13.2-3 haplotypes with basal cell carcinoma: tentative delineation of an involved region using data for single nucleotide polymorphisms in two cohorts. Carcinogenesis.

[B15] Yin J, Rockenbauer E, Hedayati M, Jacobsen NR, Vogel U, Grossman L, Bolund L, Nexo BA (2002). Multiple single nucleotide polymorphisms on human chromosome 19q13.2-3 associate with risk of Basal cell carcinoma. Cancer Epidemiol Biomarkers Prev.

[B16] de Laat WL, Jaspers NG, Hoeijmakers JH (1999). Molecular mechanism of nucleotide excision repair. Genes Dev.

[B17] Viguier J, Boige V, Miquel C, Pocard M, Giraudeau B, Sabourin JC, Ducreux M, Sarasin A, Praz F (2005). ERCC1 codon 118 polymorphism is a predictive factor for the tumor response to oxaliplatin/5-fluorouracil combination chemotherapy in patients with advanced colorectal cancer. Clin Cancer Res.

[B18] Park DJ, Zhang W, Stoehlmacher J, Tsao-Wei D, Groshen S, Gil J, Yun J, Sones E, Mallik N, Lenz HJ (2003). ERCC1 gene polymorphism as a predictor for clinical outcome in advanced colorectal cancer patients treated with platinum-based chemotherapy. Clin Adv Hematol Oncol.

[B19] Ryu JS, Hong YC, Han HS, Lee JE, Kim S, Park YM, Kim YC, Hwang TS (2004). Association between polymorphisms of ERCC1 and XPD and survival in non-small-cell lung cancer patients treated with cisplatin combination chemotherapy. Lung Cancer.

[B20] Zhou W, Gurubhagavatula S, Liu G, Park S, Neuberg DS, Wain JC, Lynch TJ, Su L, Christiani DC (2004). Excision repair cross-complementation group 1 polymorphism predicts overall survival in advanced non-small cell lung cancer patients treated with platinum-based chemotherapy. Clin Cancer Res.

[B21] Whitehead CM, Winkfein RJ, Fritzler MJ, Rattner JB (1997). ASE-1: a novel protein of the fibrillar centres of the nucleolus and nucleolus organizer region of mitotic chromosomes. Chromosoma.

[B22] Schottenfeld D, Winawer SJ, Schottenfeld D and J.F.Jr. F (1996). Cancers of the large intestine. Cancer Epidemiology and Prevention.

[B23] Hecht SS, Hoffmann D (1988). Tobacco-specific nitrosamines, an important group of carcinogens in tobacco and tobacco smoke. Carcinogenesis.

[B24] Brooks PJ, Theruvathu JA (2005). DNA adducts from acetaldehyde: implications for alcohol-related carcinogenesis. Alcohol.

[B25] Melikian AA, Sun P, Prokopczyk B, El-Bayoumy K, Hoffmann D, Wang X, Waggoner S (1999). Identification  of benzo[a]pyrene metabolites  in cervical mucus and DNA adducts in cervical tissues in humans by gas chromatography-mass spectrometry. Cancer Lett.

[B26] Benhamou S, Laplanche A, Guillonneau B, Mejean A, Desgrandchamps F, Schrameck C, Degieux V, Perin F (2003). DNA adducts in normal bladder tissue and bladder cancer risk. Mutagenesis.

[B27] Fang JL, Vaca CE (1997). Detection of DNA adducts of acetaldehyde in peripheral white blood cells of alcohol abusers. Carcinogenesis.

[B28] Tjonneland A, Gronbaek M, Stripp C, Overvad K (1999). Wine intake and diet in a random sample of 48763 Danish men and women. Am J Clin Nutr.

[B29] Storm HH, Michelsen EV, Clemmensen IH, Pihl J (1997). The Danish Cancer Registry--history, content, quality and use. Dan Med Bull.

[B30] Miller SA, Dykes DD, Polesky HF (1988). A simple salting out procedure for extracting DNA from human nucleated cells. Nucleic Acids Res.

[B31] Laska MJ, Nexo BA, Vistisen K, Poulsen HE, Loft S, Vogel U (2005). Polymorphisms in RAI and in genes of nucleotide and base excision repair are not associated with risk of testicular cancer. Cancer Lett.

[B32] Barlow WE, Ichikawa L, Rosner D, Izumi S (1999). Analysis of case-cohort designs. J Clin Epidemiol.

[B33] Barlow WE (1994). Robust variance estimation for the case-cohort design. Biometrics.

[B34] Greenland S (1995). Dose-response and trend analysis in epidemiology: alternatives to categorical analysis. Epidemiology.

[B35] Vogel U, Olsen A, Wallin H, Overvad K, Tjonneland A, Nexo BA (2005). Effect of polymorphisms in XPD, RAI, ASE-1 and ERCC1 on the risk of basal cell carcinoma among Caucasians after age 50. Cancer Detect Prev.

[B36] Gondal G, Grotmol T, Hofstad B, Bretthauer M, Eide TJ, Hoff G (2005). Lifestyle-related risk factors and chemoprevention for colorectal neoplasia: experience from the large-scale NORCCAP screening trial. Eur J Cancer Prev.

