# Supplementary material for: A haplotype of polymorphisms in ASE-1, RAI and ERCC1 and the effects of tobacco smoking and alcohol consumption on risk of colorectal cancer: a danish prospective case-cohort study
Source: BMC Cancer. 2008 Feb 20;8:54. doi: 10.1186/1471-2407-8-54 (PMC2263058; doi:10.1186/1471-2407-8-54)
Supplement: Additional File 1 — Table 4 Gene-environment interaction stratified by gender. ERCC1 Asn118Asn, ASE-1 G-21A and RAI IVS1 A4364G genotype-specific effects of average smoking intensity and consumption of alcohol on the risk of colorectal cancer. The table presents the genotypic distribution of the three polymorphisms in the ERCC1, ASE-1 and RAI genes for both cases and controls, and the incidence rate ratios of colorectal cancer risk associated with the three polymorphisms. [file 1471-2407-8-54-S1.pdf]

Additional Table 4: Gene-environment interaction stratified by gender

ERCC1 Asn118Asn, *ASE-1* G-21A and *RAI* IVS1 A4364G genotype-specific effects of average smoking intensity and consumption of alcohol on the risk of colorectal cancer

		ERCC1 Asn118Asn							
		Men				Women			
	SNP	N cases/sub-cohort	IRR[a]	CI (95%)[b]	P[c]	N cases/sub-cohort	IRR[a]	CI (95%)[b]	P[c]
Smoking intensity[d] (per 10g tobacco/day)	TT	67 / 122	0.99	0.73-1.33		45 / 88	1.55	0.85-2.84	
	CT	78 / 145	0.92	0.73-1.16	0.82	37 / 84	1.83	1.07-3.14	0.16
	CC	30 / 40	1.02	0.74-1.40		11 / 31	0.91	0.46-1.80	
	missing	3 / 7				4 / 2			
Alcohol intake[e] (per 10g/day)	TT	82 / 171	1.03	0.91-1.15		79 / 154	0.92	0.69-1.23	
	CT	99 / 188	1.11	1.02-1.21	0.15	64 / 154	1.15	0.94-1.41	0.25
	CC	34 / 60	0.91	0.75-1.10		26 / 50	1.28	0.97-1.70	
	missing	3 / 11				6 / 4			

		ASE-1 G-21A							
		Men				Women			
	SNP	N cases/sub-cohort	IRR[a]	CI (95%)[b]	P[c]	N cases/sub-cohort	IRR[a]	CI (95%)[b]	P[c]
Smoking intensity[d] (per 10g tobacco/day)	GG	123 / 214	0.99	0.80-1.22		65 / 132	1.65	0.97-2.80	
	AG	47 / 89	0.92	0.68-1.24	0.19	21 / 64	1.22	0.69-2.16	0.38
	AA	6 / 5	8.16	0.77-86.29		7 / 7	2.53	0.70-9.14	
	missing	2 / 6				4 / 2			
Alcohol intake[e] (per 10g/day)	GG	150 / 290	1.05	0.98-1.14		117 / 244	1.09	0.91-1.31	
	AG	59 / 121	1.11	0.96-1.27	0.20	44 / 104	1.14	0.88-1.47	0.33
	AA	7 / 10	0.76	0.52-1.12		8 / 11	0.30	0.05-1.69	
	missing	2 / 9				6 / 3			

		RAI IVS1 A4364G							
		Men				Women			
	SNP	N cases/sub-cohort	IRR[a]	CI (95%)[b]	P[c]	N cases/sub-cohort	IRR[a]	CI (95%)[b]	P[c]
Smoking intensity[d] (per 10g tobacco/day)	AA	131 / 202	1.02	0.82-1.26		58 / 137	1.31	0.80-2.14	
	AG	39 / 97	0.86	0.62-1.18	0.53	32 / 60	1.83	1.03-3.25	0.44
	GG	5 / 9	0.86	0.50-1.48		3 / 6	1.04	0.30-3.60	
	missing	3 / 6				4 / 2			
Alcohol intake[e] (per 10g/day)	AA	153 / 270	1.08	0.99-1.19		100 / 226	1.06	0.89-1.27	
	AG	56 / 136	1.05	0.95-1.15	0.55	60 / 122	1.25	0.94-1.66	0.24
	GG	6 / 13	0.81	0.47-1.39		8 / 10	0.64	0.29-1.38	
	missing	3 / 11				7 / 4			

a) IRR: incidence rate ratio

b) CI: 95% confidence interval

c) P: test for interaction

d) Risk estimates for ever smokers only. Adjusted for smoking status (present/former/never), intake of alcohol, fruits/vegetables, fish/poultry, red and processed meat, dietary fibres, BMI and hormone replacement therapy

e) Risk estimates for alcohol consumers only. Adjusted for average smoking intensity, fruits/vegetables, fish/poultry, red and processed meat, dietary fibres, BMI and hormone replacement therapy
